# The Relationship of the Pathogenic Variant rs721048 in the Intron of the *EHBP1* Gene with the Development of Prostate Cancer and Colorectal Cancer in the Kazakh Population

**DOI:** 10.3390/genes16020171

**Published:** 2025-01-28

**Authors:** Marina Romanova, Saltanat Abdikerim, Kaisar Dauyey, Ziyo Gassanov, Nurlan Baltayev, Shyngys Satymbayev, Aigul Zhunussova, Dilyara Kaidarova, Gulnur Zhunussova

**Affiliations:** 1 Laboratory of Molecular Genetics, RSE “Institute of Genetics and Physiology” of the SC MSHE RK, Al-Farabi Ave., 93, Almaty 050060, Kazakhstan; m82p@yandex.ru (M.R.); abdikerimse@gmail.com (S.A.); k.dauyey.bio.nu@gmail.com (K.D.); aigul700@mail.ru (A.Z.); 2 Department of Molecular Biology and Genetics, Al-Farabi Kazakh National University, Almaty 050040, Kazakhstan; 3 Department of Medicine, School of Medicine, Nazarbayev University, Astana 010000, Kazakhstan; 4 Kazakh Institute of Oncology and Radiology, Almaty 050000, Kazakhstan; ziegasanov@mail.ru (Z.G.); dilyara.kaidarova@gmail.com (D.K.); 5 Almaty Oncology Centre, Almaty 050000, Kazakhstan; nurlan.baltayev@mail.ru (N.B.); shyngyssatymbaev@gmail.com (S.S.); 6 Oncology Department, Asfendiyarov Kazakh National Medical University, Almaty 050000, Kazakhstan

**Keywords:** prostate cancer, next-generation sequencing, pathogenic variant, rs721048, *EHBP1*, colorectal cancer

## Abstract

Background: Prostate cancer (PC) is one of the most common oncological diseases among men. Up to 20% of PC cases are associated with hereditary risks or syndromes. The impact of common variants, particularly *EHBP1* c.1185+30064G>A rs721048, on developing PC and other malignancies remains unclear. There are also no data on the frequency of this variant in the Kazakh population or its association with PC, nor its potential connection with other malignancies, particularly colorectal cancer (CRC). Methods: We utilized the TruSight Cancer Sequencing Panel to assess pathological genomic variants in 72 male patients with histologically verified aggressive PC and 119 patients of Kazakh nationality with histologically confirmed CRC compared to the control group. Results: A variant in the intron of the *EHBP1* gene c.1185+30064G>A rs721048 was identified in 18 patients (25%) out of 72 with PC, while in the control group of 41 healthy males, the rs721048 variant was found in only 4 (9.8%) individuals. In the CRC group, rs721048 was detected in 17 cases (14.2%) and eight control individuals (10%). Conclusions: The frequency of the *EHBP1* c.1185+30064G>A rs721048 variant in the PC group was significantly higher (*p* < 0.05) than in healthy males of Kazakh nationality. Identifying *EHBP1* c.1185+30064G>A among the male population of Kazakhstan will help form the high-risk groups for PC to prevent the development of malignant neoplasms. The presence of rs721048 was not significantly associated with the risk of developing CRC in our study.

## 1. Introduction

Prostate cancer (PC) is one of the most prevalent oncological diseases among men in terms of both incidence and mortality. More than 1.4 million people are diagnosed with PC for the first time each year, and the number of new cases is increasing annually [1]. PC can develop as part of hereditary cancer syndromes as either a primary or secondary tumor, with up to 20% of PC cases associated with hereditary risks or syndromes [2,3]. While age, race, and family history remain key risk factors, advances in genetic research have illuminated the role of inherited mutations in the pathogenesis of prostate cancer. Among the most significant are mutations in DNA repair genes such as *BRCA1*, *BRCA2*, and *ATM*, which are not only associated with hereditary breast and ovarian cancer but are also increasingly linked to prostate cancer, particularly in more aggressive forms of the disease [4]. Additionally, several studies have demonstrated the involvement of the *EHBP1* gene in malignant processes. For instance, the frequently occurring variant in the intron of the *EHBP1* gene c.1185+30064G>A rs721048 is a risk factor for developing PC in various populations [5,6]. Increased expression of the *EHBP1* gene tends to affect survival rates in patients with colorectal cancer (CRC) [7], potentially via alterations to the response to neoadjuvant chemotherapy [8]. The impact of the rs721048 variant on carcinogenesis remains contradictory. The frequency of this variant among PC patients in the Kazakh population has yet to be studied. No studies have investigated the possible connection between the rs721048 variant and the risk of developing other malignant neoplasms. Given the limited understanding of the role of this gene in malignant neoplasms, we conducted a study on the association of rs721048 with the risk of developing PC and CRC in the Kazakh population. By emphasizing the unique characteristics of the Kazakh population, we aim to contribute valuable insights while encouraging broader research into the genetic underpinnings of PC. Given the limited understanding of the role of this gene in malignant neoplasms, we conducted a study to investigate the association of rs721048 with the risk of developing PC. Furthermore, we expanded our research by including patients with CRC from the Kazakh population. The CRC patient data were obtained from our previous study [9], conducted at our research center and completed in 2023. We performed a bioinformatics re-analysis, identifying patients with and without the pathogenic variant *EHBP1* gene c.1185+30064G>A, evaluating the risk of developing PC and CRC accordingly.

## 2. Materials and Methods

The study included 72 Kazakh patients with a histologically verified diagnosis of aggressive PC. Tumor histological classification and staging were performed according to the TNM system. All patients received treatment at the Kazakh Institute of Oncology and Radiology (KazIOR). The criterion for selecting aggressive disease forms was the Gleason score (≥7) and Grade Group 3 and higher. We excluded patients with a Gleason score of 7 (3 + 4) (Grade Group 2) from the study, as a more favorable disease prognosis characterizes this group. Active surveillance is acceptable for some patients, mainly when the percentage of tumor tissue with a Grade 4 component is less than 10%. In contrast, our study focuses on patients with more aggressive forms of the disease [10]. Gleason 4 + 3 prostate cancer is agreed to be more aggressive than 3 + 4 due to the predominance of poorly differentiated grade 4 tumor cells, which are associated with worse outcomes [11]. Based on the biopsy material, Gleason scores may underestimate the accurate Gleason score found at radical prostatectomy [12]. Inclusion criteria for the study were primary tumor localization in the prostate, availability of complete data on disease stage, histological examination results, patient age, and the presence of informed consent from the patient.

A control group of 41 male individuals of Kazakh nationality without a cancer diagnosis and with a confirmed absence of malignancy was also established. Inclusion criteria for the control group were age 50 and older, without inflammatory prostate diseases, and no history of oncological diseases.

Additionally, 119 patients of Kazakh nationality with histologically confirmed CRC were selected. The patient information and staging values in the CRC group were obtained from a study conducted at our research center and completed in 2023. All patients in this group had primary tumors in the colon. Disease staging was performed according to the TNM classification. Informed consent for participation in the study was obtained from all patients. A control group for CRC patients was established, consisting of 78 individuals of Kazakh nationality without a CRC diagnosis. Both male and female patients were included in the comparison group to assess the association of rs721048 variants and the development of CRC due to the involvement of *EHBP1* in other forms of cancer affecting both males and females [13].

DNA Preparation and Sequencing

According to the manufacturer’s protocol, DNA was extracted from peripheral blood using the ReliaPrep™ Blood gDNA Miniprep System (Promega, Madison, WI, USA). Libraries from the obtained DNA were prepared using the Illumina DNA Prep kit. Enrichment was performed using the TruSight Rapid Capture Kit (Illumina, San Diego, CA, USA) with the TruSight Cancer Sequencing Panel (Illumina, USA). The concentration of the obtained DNA libraries was determined using a Qubit 2.0 fluorometer (Invitrogen, Carlsbad, CA, USA), and quality control was conducted using an Agilent Bioanalyzer 2100 (Agilent Technologies, Santa Clara, CA, USA). Paired-end high-throughput sequencing was performed on the MiSeq platform (Illumina, USA).

Bioinformatics Data Processing

Data analysis was conducted using MiSeq Reporter software v.3.0 (Illumina). All variants with ≤10 reads were filtered out. Reference genome mapping (hg19, GRCh37.p13) was performed using the Burrows–Wheeler Aligner (BWA) algorithm. Variant calling for specific genomic regions was done using the Genome Analysis Toolkit (GATK, Broad Institute, Cambridge, MA, USA). Annotation of genetic variants was performed according to the nomenclature of the Human Genome Variation Society (HGVS). Sequencing results, presented as aligned sequences in VCF file format, were analyzed using software v3.0 such as Variant Studio, SnpEff, SnpSift, and OpenCravat.

A Q-score of 30, reflecting an error rate of 1:1000, was established as the quality threshold for sequencing. The following filtering criteria were applied to eliminate low-quality nucleotide variants: read depth exceeding 50×, alternatively read depth greater than 20×, and a quality score higher than 100. Genetic variants were annotated following the HGVS nomenclature [14]. Identified variants were interpreted using ClinVar, Single Nucleotide Polymorphism Database (dbSNP), and LOVD. Genetic variants were visualized using the Integrative Genomics Viewer (IGV 2.17.4). Variants identified in the analysis were categorized based on the guidelines provided by the American College of Medical Genetics and Genomics (ACMG).

Statistical analysis was conducted using the Wilcoxon rank-sum test for continuous variables and the chi-square test for categorical variables. Results were deemed statistically significant at a threshold of *p* < 0.05.

## 3. Results

### 3.1. Characteristics of Patient and Control Groups

The first group consisted of 72 patients of Kazakh nationality with prostate cancer (PC). This group included patients with the identified pathogenic variant rs721048 and patients without pathogenic/possibly pathogenic variants. The Gleason score assessment was as follows: 12 patients had a score of 7 (4 + 3), 24 patients had a score of 8 (4 + 4 in 23 patients, 5 + 3 in 1 patient), 15 patients had a score of 9 (4 + 5 in 8 patients, 5 + 4 in 6 patients), and three patients had a score of 10 (5 + 5). The average age of the patients was 68.7 years (ranging from 50 to 92 years). At the time of diagnosis, two patients were confirmed to have stage I, 18 patients had stage II, 14 patients had stage III, and 38 patients had stage IV disease. The average age of patients with stage I disease was 66.5 years (63–70 years), with stage II was 67.2 years (ranging from 50 to 72 years), the average age of patients with stage III disease was 71.6 years (ranging from 60 to 84 years), and with stage IV was 68.2 years (ranging from 50 to 92 years). Two patients had a significant oncological history, while 12 patients had no significant history, and data were unavailable for 58 patients. The second group included 43 asymptomatic men of Kazakh nationality. The average age of the control group was 65.1 years (ranging from 50 to 80 years). Additionally, a group of patients with colorectal cancer (CRC) was established, consisting of 119 patients. This group included patients with the rs721048 variant and patients without the pathogenic variant. Patients with other pathogenic variants associated with the development of malignant tumors were not included in this study. The average age of patients in this group was 42.2 years (ranging from 21 to 72). The gender distribution was 56 (47%) men and 63 (53%) women. The distribution by disease stage was as follows: stage 0—1 patient, age 40 years; stage I—7 patients, average age 41.9 years (36–69); stage II—50 patients, average age 42.3 years (27–60); stage III—49 patients, average age 42.2 years (21–72); stage IV—12 patients, average age 35.9 years (24–49). A family history of cancer was present in 26 patients. Five patients had no family history of cancer, and the rest of the dataset had no available mention of cancer family history for 88 patients. The control group for CRC patients consisted of asymptomatic men and women, including 78 individuals. The average age of this control group was 55.1 years (ranging from 37 to 80). The gender distribution was as follows: 41 men (52.5%) and 37 women (47.5%) (Table 1).

### 3.2. rs721048 Variant Analysis

We identified a variant in the intron of the *EHBP1* gene c.1185+30064G>A (rs721048) in 18 out of 72 prostate cancer patients, accounting for 25% of the total number of patients. This variant was classified in the ClinVar database as pathogenic and is associated with an increased risk of developing prostate cancer, as well as with a more aggressive disease course. We checked the 1000 Genome Project allele frequency report on the variant rs721048, and on average, 9% of the healthy subjects had this variant [15]. This indicates the overall variability and pathogenicity role of the rs721048 variant across global populations. Patients with other pathogenic variants related to the development of oncological diseases were not included in this study. The average age of patients with the rs721048 variant was 67.1 years (ranging from 50 to 81 years). The average age of patients without the variant was 69.2 years (ranging from 50 to 92 years). The distribution by stage for patients with the rs721048 variant was as follows: 5 patients had stage II, with an average age of 70 years (59–81); 2 patients had stage III, with an average age of 67 years (60–74); and 11 patients had stage IV, with an average age of 65.8 years (50–76). The Gleason score distribution was as follows: score of 7 (4 + 3)—9 patients, mean age 66.1 years (range 59–76); score of 8 (4 + 4)—7 patients, mean age 69.7 years (range 50–81); score of 10 (5 + 5)—2 patients, mean age 62.5 years (range 55–70). The distribution by stage for patients without the rs721048 variant was as follows: stage I—2 patients (66.5 years (63–70)); stage II—13 patients (66.2 years (50–82)); stage III—12 patients (72.4 years (61–84)); and stage IV—27 patients (69.5 years (55–92)). The Gleason scores were as follows: 7 (4 + 3)—12 patients, mean age 66.2 years (range 55–73); 8 (4 + 4)—23 patients, mean age 68.1 years (range 55–84); 8 (5 + 3)—1 patient, aged 82 years; 9 (4 + 5)—8 patients, mean age 71.8 years (range 61–92); 9 (5 + 4)—7 patients, mean age 73.0 years (range 69–77); 10 (5 + 5)—3 patients, mean age 69.3 years (range 50–79).

In the control PC group, a pathogenic variant was identified in 4 (9.3%) of the 43 individuals, with an average age of 66.8 years (61–78). The average age of individuals without rs721048 was 65.1 years (ranging from 50 to 80).

In the group of patients with CRC, 17 patients (14.3%) had the rs721048 variant. The average age of patients with the variant was 41.1 years (24–69); without the variant, it was 42.3 years (21–72). The distribution by stage for patients with the variant was as follows: stage I—3 patients, average age 52.5 years (36–69); stage II—6 patients, average age 42.7 years (36–51); stage III—7 patients, average age 39.7 years (25–46); and stage IV—2 patients, average age 30.0 years (24–36). The distribution by stage for patients without the rs721048 variant was as follows: 1 patient with stage 0 (40 years); 5 patients with stage I (53.2 years (45–65)); 44 patients with stage II (42.2 years (27–60)); 42 patients with stage III (42.6 years (21–72)); and 11 patients with stage IV (37.1 years (28–49)).

In the control CRC group, eight individuals (average age 56.8 years (41–78)) had the rs721048 variant, which accounted for 10.8%. The variant was not detected in 70 individuals. The average age of individuals without the rs721048 variant was 54.8 years (37–80) (Table 2).

### 3.3. Statistical Analysis

We found no significant difference in age or gender (*p* > 0.05) between patients with and without the variant. Additionally, there was no significant difference in the frequency of rs721048 between the colorectal cancer group (119 patients) and the control group (78 individuals) (*p* = 0.41). No statistically significant differences were found in the frequency of rs721048 between the colorectal cancer group and the prostate cancer group (72 patients) (*p* = 0.06). However, a statistically significant difference (*p* < 0.05) was identified in the frequency of this variant between the prostate cancer group and the group of healthy male individuals (43 individuals). The odds ratio between rs721048 and prostate cancer risk was measured using OR and was 3.25 (95% CI 1.02–10.36) (Table 3).

## 4. Discussion

We identified a pathological variant in the *EHBP1* gene (rs721048) in 18 (25%) of Kazakh prostate cancer patients. According to the current literature, this variant is associated with an increased risk and earlier onset of prostate cancer [16]. Additionally, this variant was found in four healthy Kazakh male individuals (9.8%). The frequency of this variant in the non-cancer group was significantly lower (*p* = 0.05). Furthermore, the rs721048 variant was detected in 17 (14.2%) Kazakh patients with colorectal cancer. Among healthy control individuals without a CRC diagnosis, the frequency of rs721048 occurrence was 10.8%, consistent with global data on the frequency of this variant in Asian populations [17]. However, a study from Iran focused on 103 patients with prostate cancer and 100 patients with benign prostatic inflammatory diseases demonstrated no significant association between the rs721048 polymorphism and prostate cancer [18].

**Figure 1:**
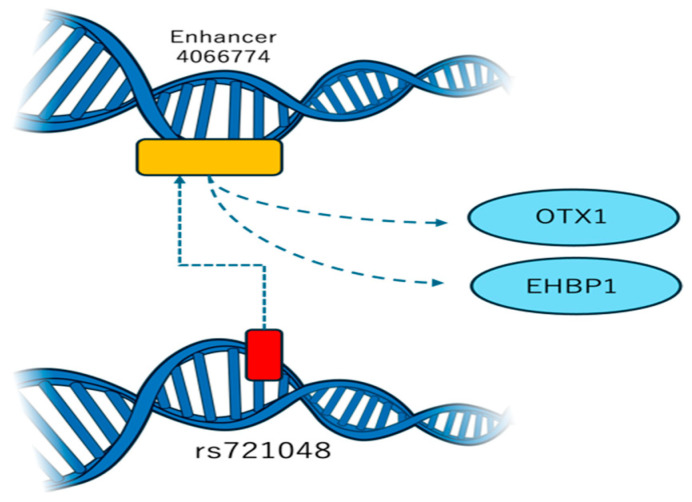
Interaction between rs721048 and the enhancer in the spatial genome model, red label indicates the relative position of the study variant, yellow rectangle stands for the relative position of the OTX1 and EHBP1 genes on chromosome 2p15. Illustration based on NIAID NIH BIOART source (bioart.niaid.nih.gov/bioart/124 accessed on 1 December 2024).

The *EHBP1* gene is located at the human chromosome 2; it encodes a protein involved in endocytic transport, attracting members of the oncogene family Rab8 and Rab10 [19]. It links endocytic vesicles to the actin cytoskeleton and plays a key role in modulating the glucose transporter 4 in adipocytes [20]. The Rab10-EHBP1-EHD2 complex forms a trimer crucial in lipid phagocytosis by hepatocytes and autophagic digestion of lipid droplets [21]. Molecular pathways affected by *EHBP1* include tissue homeostasis and embryonic development associated with intracellular transport mediated by *Wingless*/*Wnt* signaling [22]. Alternative splicing regulated by the *MYC* family of transcription factors can be aberrant in colorectal cancer [23], affecting *EHBP1* responsible for cellular organization in cancer cells [24]. Additionally, three-dimensional genome modeling demonstrated that the rs721048 variant promotes interaction with a chromatin segment containing an active enhancer that regulates two genes: *EHBP1* and *OTX1* [25]. This interaction may represent a potential connection to PC development, as depicted in Figure 1.

The *OTX1* gene is located on the human chromosome 2 (2p13) [26]. The *OTX1* protein acts as a transcription factor, critical in brain regionalization, structural formation, and sensory organ development during embryogenesis [27]. Recent studies have highlighted the role of *OTX1* expression in carcinogenesis [28]. The literature describes *OTX1* overexpression in tumor tissues across various cancers, including colorectal [29], breast [30], bladder [31], thyroid [32], and cervical [33] malignancy. Elevated *OTX1* expression promotes cell proliferation and tumor growth, suggesting that *OTX1* could be a potential oncogene and a target for anticancer therapy [34].

Due to the observation of *OTX1* and *EHBP1* expression in various tumor tissues across cancer types, we hypothesized that the rs721048 variant, through enhancer activation, might increase the risk of malignancies beyond prostate cancer. Consequently, we analyzed and compared the data from a CRC patient group to the control group. Among the colorectal cancer patients, 17 (14.2%) were found to have the c.1185+30064G>A variant in the *EHBP1* gene; patients with other pathogenic variants in genes included in our panel were excluded. In the control group of individuals without a cancer diagnosis, 8 (10.2%) had the same variant. However, these two groups had no statistically significant difference in variant frequency.

Therefore, based on our findings, we conclude that the frequency of the pathogenic *EHBP1* c.1185+30064G>A variant in Kazakh individuals without cancer is approximately 10%, which aligns with global population data. While our study provides valuable insights into the genetic landscape of prostate cancer within the Kazakh population, we acknowledge the need for further validation to strengthen the generalizability of our findings. Future studies should include diverse populations to determine whether the observed genetic modification is consistent across ethnic and geographic groups. Our study demonstrated that the frequency of this variant is significantly higher among Kazakh individuals with PC compared to the male control group. However, further research with a larger cohort is necessary for more substantial evidence of disease progression and pathogenesis of the rs721048 variant in PC. Although the variant is in a non-coding genome region, we hypothesize that it may exert its effect through interaction with enhancer activating genes involved in a wide range of malignancies, as other non-coding RNA elements have been linked with the development of PC [35]. Our analysis of the pathogenic c.1185+30064G>A variant frequency in the *EHBP1* gene in CRC and groups of individuals without a cancer diagnosis of both genders revealed no significant difference between these groups.

Our findings may not be fully generalizable to other populations due to the study’s focus on a Kazakh cohort and the specific genetic variant rs721048. PC risk is influenced by both genetic and environmental factors, which can vary significantly across different ethnic and geographic groups as well as on the molecular level. The frequency and impact of rs721048 may differ in other populations, potentially affecting the applicability of our results. Future studies should validate these findings in diverse cohorts to better understand the role of rs721048 and other genetic variants in prostate cancer risk across populations.

## 5. Conclusions

The frequency of *EHBP1* c.1185+30064G>A in the Kazakh population is consistent with international databases. The presence of this pathogenic variant significantly increases the risk of PC. Detecting the *EHBP1* c.1185+30064G>A variant in the clinical setting may indicate early-onset and aggressive PC that clinicians should consider for proper diagnosis and management. The pathogenic rs721048 variant did not demonstrate an increased risk of CRC in our study population despite the literature reports on the association between rs721048 and enhancer activity specific to *EHBP1* and *OTX1* genes.

## Figures and Tables

**Table 1:** Characteristics of patient and control groups.

	Patients with Prostate Cancer	Control Group, Male	Patients with Colorectal Cancer	Control Group, Male and Female
Number	72	43	119	78
Age, years	68.7 (50–92)	65.1(50–80)	42.2 (21–72)	55.1 (37–80)
Stage:				
0, number	0	-	1	-
Age, years	-		40	
I, number	2	-	7	-
Age, years	66.5 (63–70)		41.9 (36–69)	
II, number	18	-	50	-
Age, years	67.2 (50–82)		42.3 (27–60)	
III, number	14	-	49	-
Age, years	71.6 (60–84)		42.2 (21–72)	
IV, number	38	-	12	-
Age, years	68.2 (50–92)		35.9 (24–49)	
History:				
With family history	2	-	26	78
Without family history	12	-	5	-
No data	58	43	88	
Gender:				
Male	72	43	56	41
Female	0	0	63	37
Gleason score				
7 (4 + 3) Age, years	21 66.2 (55–76)	-	-	-
8 (4 + 4) Age, years	29 68.6 (50–84)	-	-	-
8 (5 + 3) Age, years	1 82	-	-	-
9 (4 + 5) Age, years	8 71.8 (61–92)	-	-	-
9 (5 + 4) Age, years	7 73.0 (69–77)	-	-	-
10 (5 + 5) Age, years	5 66.6 (50–79)	-	-	-

**Table 2:** Distribution of patients and individuals without a cancer diagnosis with and without rs721048 by age, family history, and disease stage.

	Prostate Cancer Group	Control Group	Colorectal Cancer Group	Control Group
Number	72	43	119	78
Number with rs721048	18 (25%)	4 (9.3%)	17 (14.2%)	8 (10.8%)
Without variant	54 (75%)	39 (91.7%)	102 (85.8%)	70 (89.2%)
Age of patients with variant, years	67.1 (50–81)	66.8 (61–78)	41.1 (24–69)	56.8 (41–78)
Age of patients without variant, years	69.2 (50–92)	65.1 (50–80)	42.3 (21–72)	54.8 (37–80)
Patients with variant, family history:				
With family history	0	-	7	-
Without family history	1	-	1	-
No data	17	4	9	8
Patients without variant, Family History:				
With family history	2	-	19	-
Without family history	11	-	4	-
No data	21	39	79	70
Stage: patients with variant				
I, number	0	-	3	-
Age, years	-	-	52.5 (36–69)	-
II, number	5	-	6	-
Age, years	70 (59–81)	-	42.7 (36–51)	-
III, number	2	-	7	-
Age, years	67 (60–74)	-	39.7 (25–46)	-
IV, number	11	-	2	-
Age, years	65.8 (50–76)	-	30.0 (24–36)	-
Gleason score				
7 (4 + 3) Age, years	9 66.1 (59–76)	-	-	-
8 (4 + 4) Age, years	7 69.7 (50–81)	-	-	-
8 (5 + 3) Age, years	0	-	-	-
9 (4 + 5) Age, years	0	-	-	-
9 (5 + 4) Age, years	0	-	-	-
10 (5 + 5) Age, years	2 62.5 (55–70)	-	-	-
Gender:				
Male	18	4	8	4
Female	-	-	9	4
Stage: patients without variant				
0, number	0	-	1	-
Age, years		-	40	-
I, number	2	-	5	-
Age, years	66.5 (63–70)	-	53.2 (45–65)	-
II, number	13	-	44	-
Age, years	66.2 (50–82)	-	42.2 (27–60)	-
III, number	12		42	-
Age, years	72.4 (61–84)	-	42.6 (21–72)	-
IV, number	27	-	11	-
Age, years	69.5 (55–92)	-	37.1 (28–49)	
Gleason score				
7 (4 + 3) Age, years	12 66.2 (55–73)	-	-	-
8 (4 + 4) Age, years	23 68.1 (55–84)	-	-	-
8 (5 + 3) Age, years	1 82	-	-	-
9 (4 + 5) Age, years	8 71.8 (61–92)	-	-	-
9 (5 + 4) Age, years	7 73.0 (69–77)	-	-	-
10 (5 + 5) Age, years	3 69.3 (50–79)	-	-	--
Gender:				
Male	54	43	48	37
Female	-	-	54	33

**Table 3:** Risk factor and age distribution in cancer and control groups.

Groups	Patients (n)	Average Age (Range)	rs721048, N	*p*-Value (Age)	*p*-Value (Gender)	*p*-Value (Risk Factor)	Odds Ratio (OR)	95% Confidence Interval (CI)
PC group	72	68.7 (50–92)	18	>0.05		<0.05	3.25	1.02–10.36
Control group (male)	43	65.1(50–80)	4					
CRC group	119	42.2 (21–72)	17		>0.05	0.41	1.00	0.595–3.557
Control group (male + female)	78	55.1 (37–80)	8		>0.05			
PC group with rs721048, age	18	67.1 (50–81)	18	>0.05				
Control group with rs721048	4	69.2 (50–92)	4	>0.05				
PC group without rs721048	54	69.2 (50–92)	54	>0.05				

## Data Availability

The data presented in this study are available on request from the corresponding author.
